# The Treatment of Sleep Disorders in Parkinson’s Disease: From Research to Clinical Practice

**DOI:** 10.3389/fneur.2017.00042

**Published:** 2017-02-16

**Authors:** Giuseppe Loddo, Giovanna Calandra-Buonaura, Luisa Sambati, Giulia Giannini, Annagrazia Cecere, Pietro Cortelli, Federica Provini

**Affiliations:** ^1^Department of Biomedical and Neuromotor Sciences, University of Bologna, Bologna, Italy; ^2^Bellaria Hospital, IRCCS Institute of Neurological Sciences of Bologna, Bologna, Italy

**Keywords:** insomnia, sleep-related movement disorders, nocturia, parasomnias, excessive daytime sleepiness

## Abstract

Sleep disorders (SDs) are one of the most frequent non-motor symptoms of Parkinson’s disease (PD), usually increasing in frequency over the course of the disease and disability progression. SDs include nocturnal and diurnal manifestations such as insomnia, REM sleep behavior disorder, and excessive daytime sleepiness. The causes of SDs in PD are numerous, including the neurodegeneration process itself, which can disrupt the networks regulating the sleep–wake cycle and deplete a large number of cerebral amines possibly playing a role in the initiation and maintenance of sleep. Despite the significant prevalence of SDs in PD patients, few clinical trials on SDs treatment have been conducted. Our aim is to critically review the principal therapeutic options for the most common SDs in PD. The appropriate diagnosis and treatment of SDs in PD can lead to the consolidation of nocturnal sleep, the enhancement of daytime alertness, and the amelioration of the quality of life of the patients.

## Introduction

Non-motor symptoms (NMSs) are present in almost all patients with Parkinson’s disease (PD) frequently preceding the onset of motor symptoms (1). NMSs usually impact on the patients’ quality of life, representing a possible cause of institutionalization (2, 3). Among NMSs, after psychiatric symptoms, sleep disorders (SDs) constitute the second most frequent complaint, affecting 64% of PD patients, ranging from 41.1% in naïve patients to 78.3% in complicated patients (1). SDs in PD are multifactorial and include nocturnal and diurnal manifestations. Reduced sleep efficiency and an increased number of awakenings characterize sleep in PD (4). These disturbances are linked, on one side, to PD motor (akinesia, rigidity, and dystonia) and autonomic symptoms (nocturia) and, on the other side, to the presence of concomitant SDs such as REM sleep behavior disorder (RBD), restless legs syndrome (RLS), or breathing disorders such as obstructive sleep apnea (OSA) (5). Diurnal manifestations include excessive daytime sleepiness (EDS) and sudden sleep attacks, which could be a consequence of nocturnal sleep impairment or dopaminergic treatment or, more interestingly, to the neurodegenerative process of PD itself dysregulating the circadian sleep–wake rhythm (4–8). Apart from instrumental tools, specific and validated scales have been designed to evaluate SDs in PD [Parkinson’s Disease Sleep Scale (PDSS) and its modified version (PDSS-2), the Scales for Outcomes in PD-Sleep (SCOPA-S)] (9, 10). Despite the importance of identifying and treating SDs in PD patients, large-scale, randomized controlled trials for treatment options in this population are lacking. Our narrative review will discuss the available optimal therapeutic strategies, based on the literature data, to use in clinical practice.

## Nocturnal SDs

### Insomnia

According to the International Classification of SDs, chronic insomnia is characterized by difficulties initiating sleep, maintaining sleep or waking up earlier than desired in the morning (Table 1) (11). Insomnia is the most frequent SD in PD patients (36.9%) with a prevalence ranging from 27 to 80%. The most frequent complaint of PD patients is maintenance insomnia with frequent awakenings and sleep fragmentation (81%), but initial (18%) and terminal insomnia (40%) are also possible (12). Video-polysomnographic studies showed an increased sleep latency and frequent and sometimes prolonged intra-sleep awakenings. The representation of the different sleep stages is physiological (4, 13). In addition to the neurodegeneration process of PD, the most important factors for developing insomnia are female gender, PD duration, and the presence of depression and anxiety. Other factors, such as cough, cold sensations, heat sensations, and pain, which are more common in PD compared to controls, are suggested as contributing factors for sleep fragmentation. Moreover, some drugs such as dopamine agonists and their withdrawal, entacapone, selegiline, and rasagiline (although literature data are conflicting) could increase the risk of insomnia if compared to placebo (14–19).

**Table 1:** **Diagnostic criteria for chronic insomnia (International Classification of Sleep Disorders)**.

Criteria A–F must be met: The patient reports, or the patient’s parent or caregiver observes, one or more of the following: Difficulty initiating sleep. Difficulty maintaining sleep. Waking up earlier than desired. Resistance to going to bed on appropriate schedule. Difficulty sleeping without parent or caregiver intervention. The patient reports, or the patient’s parent or caregiver observes, one or more of the following related to the nighttime sleep difficulty: Fatigue/malaise. Attention, concentration, or memory impairment. Impaired social, family, occupational, or academic performance. Mood disturbance/irritability. Daytime sleepiness. Behavioral problems (e.g., hyperactivity, impulsivity, and aggression). Reduced motivation/energy/initiative. Proneness for errors/accidents. Concerns about or dissatisfaction with sleep. The reported sleep/wake complaints cannot be explained purely by inadequate opportunity (i.e., enough time is allotted for sleep) or inadequate circumstances (i.e., the environment is safe, dark, quiet, and comfortable) for sleep. The sleep disturbance and associated daytime symptoms occur at least three times per week. The sleep disturbance and associated daytime symptoms have been present for at least three months. The sleep/wake difficulty is not better explained by another sleep disorder.

Instead, akinesia, bed-turning difficulties, cramps, nocturia, difficult breathing, and nightmares seem not to be direct causes of insomnia in PD (5, 13, 20).

**Figure 1:**
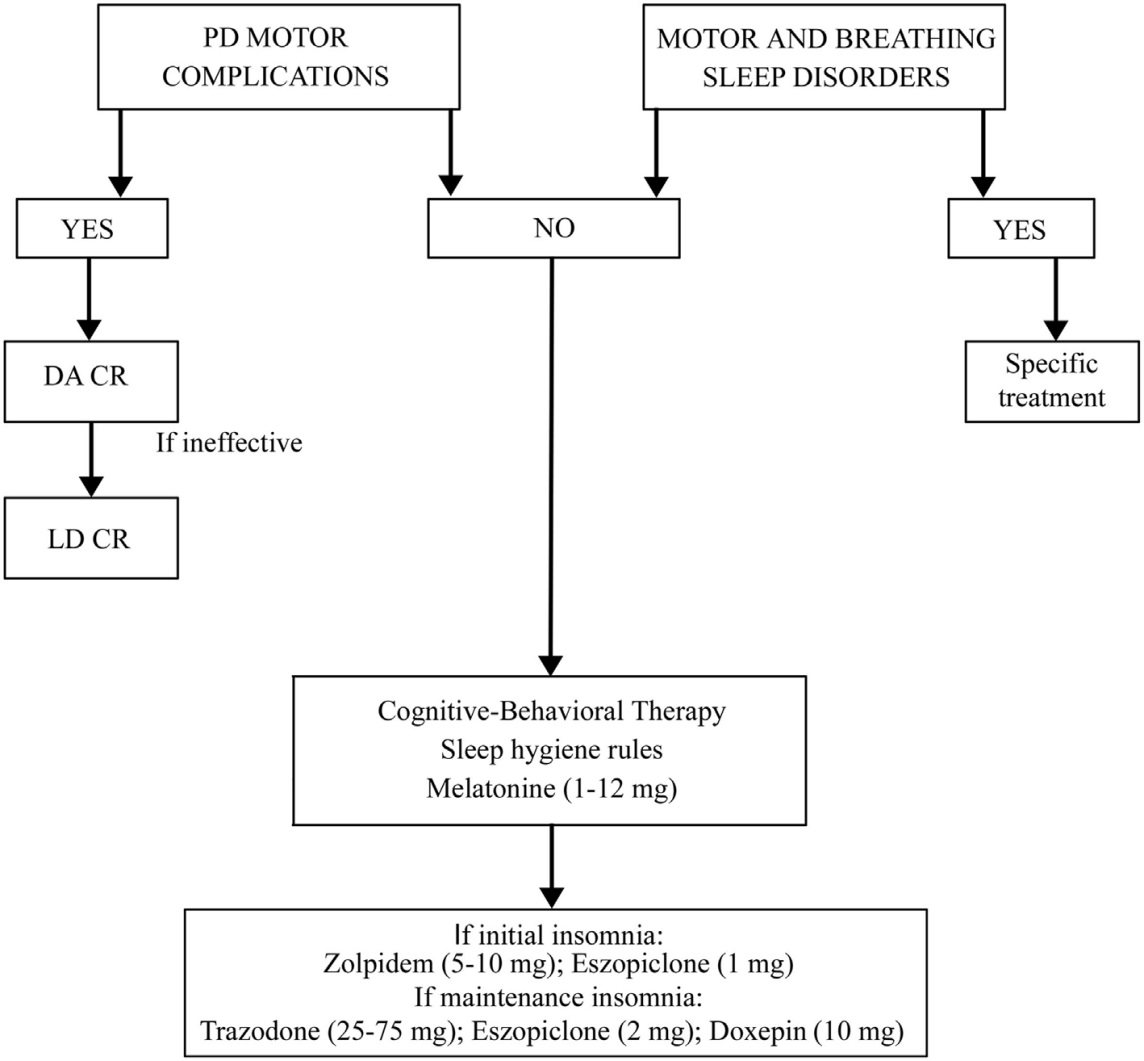
**Treatment algorithm for insomnia**.

The treatment for insomnia (pharmacological or behavioral) must be preceded by a correct identification of the type of insomnia (initial, of maintenance, or terminal) and of possible disorders and factors causing it (Figure 1). It is necessary to rule out and treat motor or sleep breathing disorders, if present. If insomnia is iatrogenic or due to motor complications of PD, it is useful to modify the therapy. Levodopa–carbidopa controlled-release (LD-CR) improves sleep-associated motor symptoms that may contribute to insomnia, although data documenting an objective improvement in sleep parameters or in sleep satisfaction are insufficient. A double-blind crossover study, comparing the effect of a single dose of 100/25 mg LD-CR with placebo, in 40 fluctuating PD patients, showed an improvement in nocturnal akinesia and only a not-significant trend of increase in total sleep time without any improvement in sleep latency and sleep fragmentation (21). In another randomized study on 32 akinetic rigid PD patients, LD-CR 200 mg before sleeping reduced nocturnal akinesia but did not significantly improve any objective sleep parameter, such as sleep latency, total sleep time, and number of arousals, compared to placebo (22).

**Figure 2:**
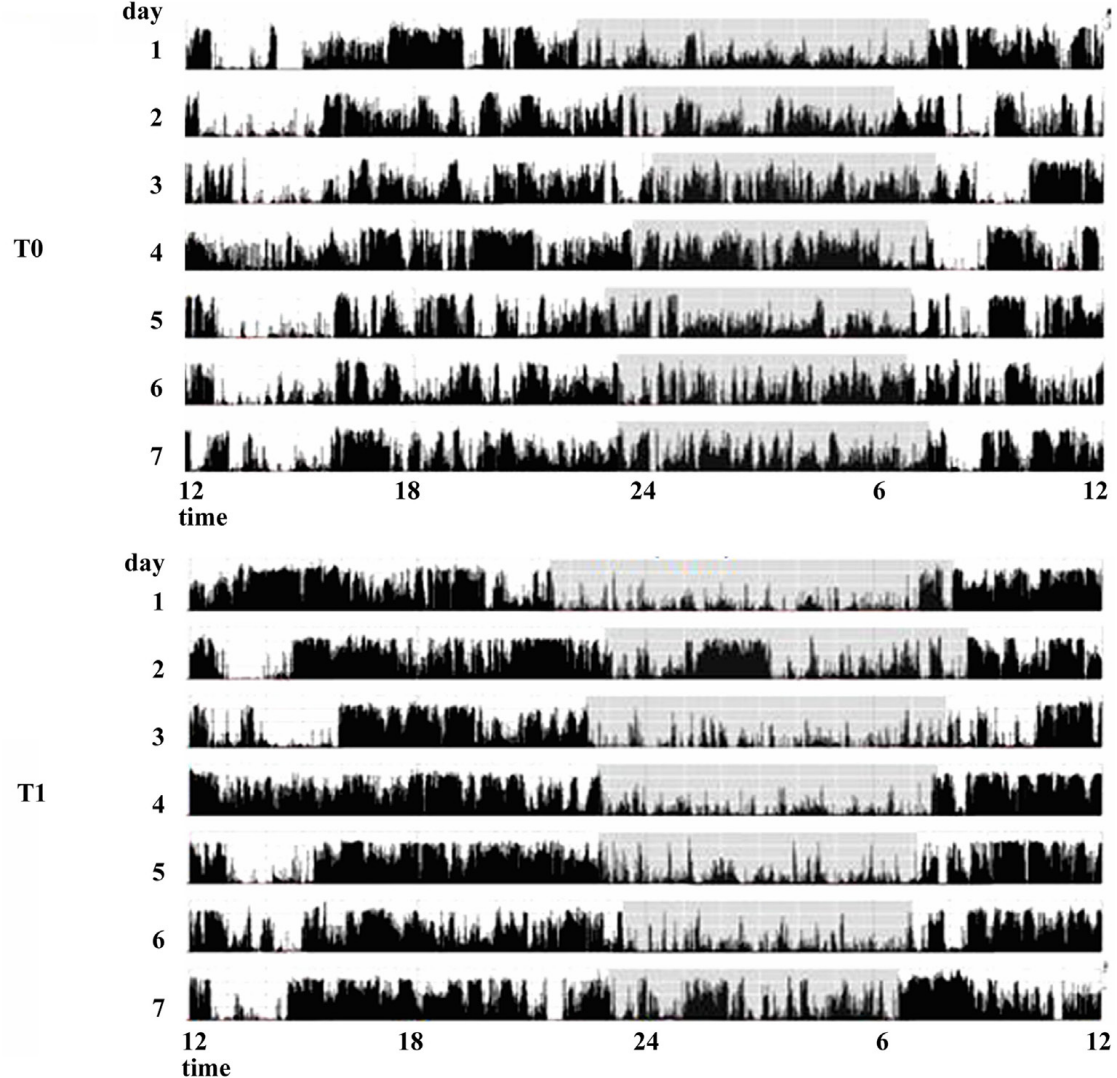
**Actigraphic recordings of a patient with Parkinson’s disease with sleep complaints in basal condition (T0) and during therapy with rotigotine 24-h transdermal patch (T1)**. During rotigotine therapy (T1), the actigraphic recording demonstrates a marked reduction in nighttime motor activity (gray stripe) compared to the basal period. A daytime reduction in diurnal naps is also noticeable (see period between 9 and 11 a.m.).

The effect of dopamine agonists (DAs) on SDs in PD has been evaluated usually as secondary endpoint in many randomized controlled trials showing an improvement in sleep parameters. Ropinirole 24-h prolonged release (2–24 mg/day) as adjunctive therapy to levodopa, significantly improved PDSS scores in 93/198 patients with motor fluctuations enrolled in a randomized, placebo-controlled study (23). A double-blind, double-dummy, randomized, placebo-controlled trial, comparing the efficacy of pramipexole (up to 4.5 mg/day) and transdermal rotigotine patch (up to 16 mg/day) on advanced-stage PD patients treated with LD showed for both drugs small, but significant, improvement in sleep parameters assessed by PDSS (24). Rotigotine transdermal patch (2–16 mg/day) improved early morning motor function and PDSS-2 scores in a multinational, randomized double-blind placebo-controlled study on 287 PD patients with unsatisfactory early morning motor symptom control and motor symptoms during the night (25). These data have been confirmed by two successive actigraphic and polysomnographic studies specifically designed to objectively evaluate the effect of rotigotine on sleep. These studies demonstrated that the rotigotine patch reduces nocturnal motor activity, duration of wake after sleep onset (WASO), nocturia, pain, and coexisting SDs such as RLS in PD patients. The rotigotine patch also reduces the number and the duration of daytime sleep episodes and improves quality of life (Figure 2) (26, 27). Finally, rasagiline, a monoamine oxidase B inhibitor, can improve insomnia probably increasing endogenous melatonin levels. A single-center, prospective, observational study compared 19 PD patients treated with LD 200–300 mg/day associated with rasagiline 1 mg/day with 19 PD patients treated only with LD 200–300 mg/day. After 12 weeks of treatment, the group treated with LD and rasagiline showed a significant reduction in mean sleep latency and an increase in mean total sleep time (28).

If insomnia is not iatrogenic and not due to PD motor complications, the principal therapy remains the cognitive behavioral therapy (29, 30). This treatment comprises advice on sleep–wake behavior hygiene, stimulus control, sleep restriction, relaxation, and cognitive techniques (20).

In the cases in which pharmacological therapy is necessary, eszopiclone, doxepin, zolpidem, trazodone, ramelteon, and melatonin could be useful although these drugs are investigational for the treatment of insomnia in PD because there is insufficient evidence regarding their efficacy. To avoid tolerance, hypnotic drugs should ideally be used for no longer than 4–5 weeks (30). Eszopiclone (2–3 mg/day) was tested in 30 PD patients with insomnia in a placebo-controlled trial. This drug does not increase total sleep time but reduces awakenings during the night and improves sleep quality. Thirteen percent of patients reported mild adverse events such as dizziness and sedation (31). In a three-arm 6-week placebo randomized pilot study in 18 PD patients, treated respectively with doxepin (10 mg daily) and cognitive behavioral therapy, doxepin improved the Pittsburgh Sleep Quality Index-Sleep Disturbances Subscale, the SCOPA-night score, the Insomnia Severity Index, the Fatigue Severity Scale, and the Montreal Cognitive Assessment. Adverse events, such as mild fatigue, transient mild nausea and unsteadiness were reported in only three cases (32). The use of zolpidem for insomnia in PD patients is still debated because randomized controlled trials in this population are lacking. Drowsiness and risk of falls are potential relevant side effects in PD (33, 34). Also, trazodone, one of the most used drugs for insomnia in elderly people with a safety profile could present possible side effects such as increased rates of falls, dizziness, impairments in short-term memory, and worsening in verbal memory (35–39). Ramelteon has been studied in PD patients with RBD improving sleep latency (40). Melatonin is established as effective in improving patients’ perception of sleep quality, but data are conflicting regarding objective improvement in sleep polysomnographic parameters (41, 42). A multi-site double-blind placebo-controlled crossover trial in 40 PD patients showed that melatonin 50 mg compared to placebo improved total sleep time, while melatonin 5 mg improved the subjective sleep disturbances perception and reduced daytime sleepiness (43). Another trial in 16 PD patients showed that melatonin 3 mg improved the subjective quality of sleep (44). Globally considering the circadian sleep–wake cycle dysregulation affecting PD patients, melatonin is effective with a good safety profile (8, 45).

Summing up, the treatment of insomnia in PD needs to rule out and eventually to treat other sleep-related motor and breathing disorders. The second step is to treat PD motor complications, if present, using prolonged release dopaminergic drugs or LD. In all the cases, it is mandatory to recommend sleep hygiene rules and cognitive-behavioral therapy, alone or in association with melatonine also at high dosages. If these measures are ineffective, a pharmacological therapy with Zolpidem, Eszopiclone, Trazodone, or Doxepine could be added (Figure 1).

### Nocturia

Nocturia affects 35% of PD patients (1). The mechanism underlying nocturia remains unclear, but it has been suggested that it may be related to autonomic modifications and to the loss of the normal D1-mediated inhibition of micturition associated with detrusor hyperreflexia (46–50).

**Figure 3:**
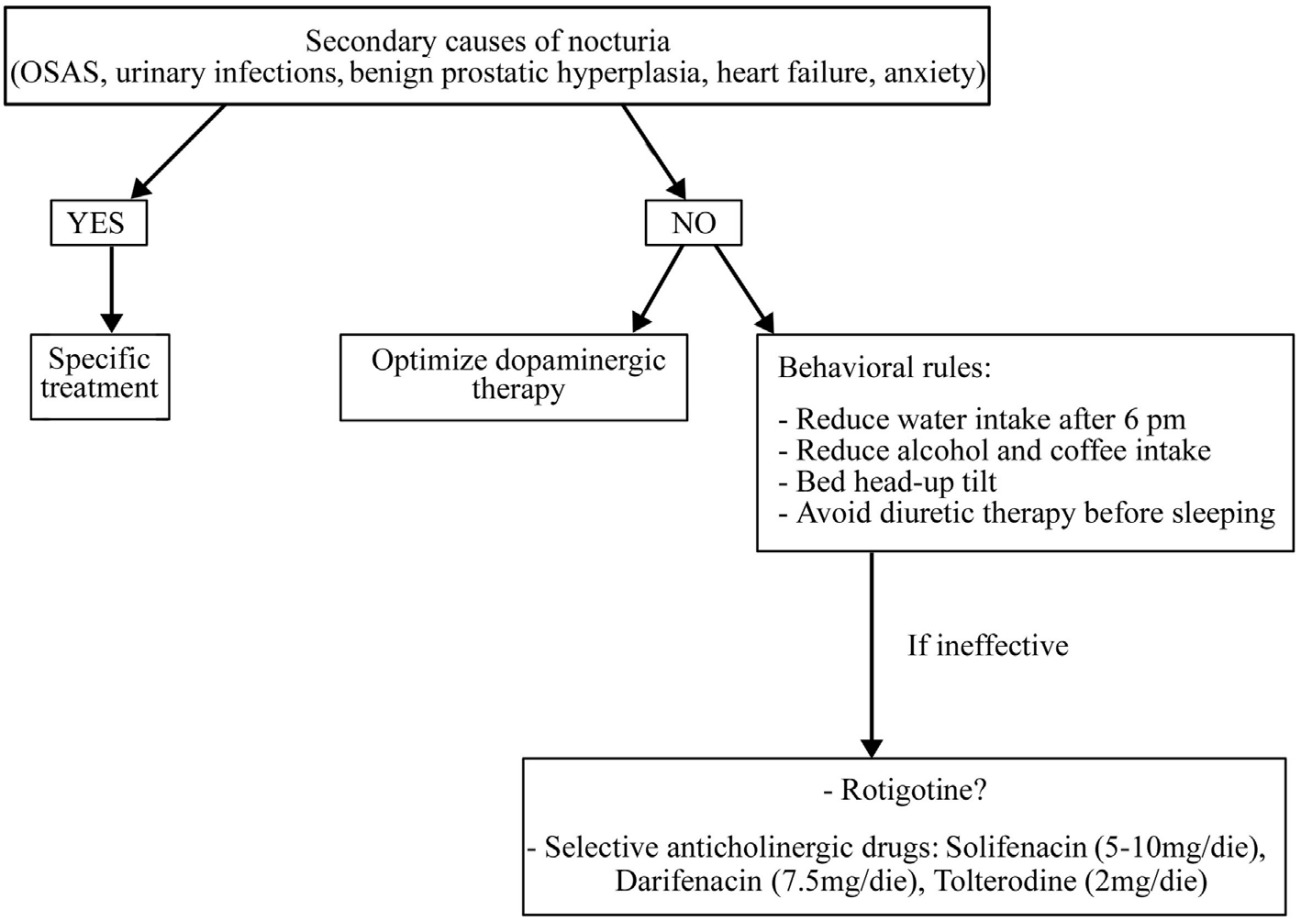
**Treatment algorithm for nocturia**.

Before starting treatment, it is necessary to rule out and treat all the secondary causes of nocturia, if present (such as OSAS, urinary infections, benign prostatic hypertrophy, heart failure, and anxiety) and optimize dopaminergic therapy (51) (Figure 3).

In addition, it is necessary to apply some behavioral rules such as reducing water intake late in the afternoon and avoid diuretic therapy before sleeping (for more details, see Figure 3). If these measures are ineffective, a pharmacological treatment could be started also if randomized controlled trials on the drug treatment of nocturia in PD patients are lacking. It is uncertain whether LD can improve micturition disorders. In a study on 26 LD-naïve PD patients, an acute challenge of LD worsened the detrusor’s overactivity while, after 2 months of chronic LD therapy, an acute challenge showed contrary results (52). Some reports have shown storage-facilitating effects of dopaminergic drugs, but there are no conclusive data regarding these drugs (50). Subcutaneous apomorphine (3–8 mg) reduced detrusor hyperreflexia in 12 PD patients without dopaminergic therapy (53). Rasagiline 1 mg incremented bladder capacity and significantly decreased residual volume in 20 mild PD patients treated with DAs (54). The efficacy of intranasal desmopressin and botulinum toxin injections into the detrusor muscle are not sufficiently proven as well as the anthicholinergic drugs, which are the most used drugs for overactive bladder in elderly people. These drugs need caution in PD patients for their side effects such as sleepiness, confusion, and cognitive impairment. Among anthicholinergic drugs, M3 receptor selective agents such as solifenacin (5–10 mg/day), darifenacin (7.5 mg/day), and tolterodine (2 mg/day) have a better tolerability than non-selective drugs such as oxybutinin (5 mg/bid) and trospium chloride (10–20 mg/bid) (50).

### Sleep-Related Movement Disorders

#### Restless Legs Syndrome

Restless legs syndrome is a sensorimotor disorder occurring in the evening or at night, characterized by an urge to move the legs usually associated with uncomfortable and unpleasant sensations in the legs relieved by movement (Table 2) (11). RLS affects 15% of patients with PD and occurs either before or after the onset of PD (1). In the *de novo* untreated populations, the prevalence of RLS seems to be similar to that of controls across populations. On the contrary, RLS prevalence increases during the course of PD and treatment duration, independent of the drug dosages (55). PD patients with RLS have higher age at PD onset, worse sleep quality, and more cardiovascular and anxiety disturbances (56, 57). Also, the presence of a secondary condition, such as iron deficiency, could explain the association between RLS and PD. The pathophysiology of RLS in PD patients is still debated but differs from that of idiopathic RLS (12). PD patients with RLS could have more preserved nigrostriatal dopaminergic pathways than those without RLS suggesting a non-linear relationship between dopaminergic dysfunction and RLS (57–60).

**Table 2:** **Diagnostic criteria for restless legs syndrome (RLS) (International Classification of Sleep Disorders)**.

Criteria A–F must be met: An urge to move the legs, usually accompanied by or thought to be caused by uncomfortable and unpleasant sensations in the legs. These symptoms must: Begin or worsen during periods of rest or inactivity such as lying down or sitting; Be partially or totally relieved by movement, such as walking or stretching, at least as long as the activity continues; and Occur exclusively or predominantly in the evening or night rather than during the day. The above features are not solely accounted for as symptoms of another medical or a behavioral condition (e.g., leg cramps, positional discomfort, myalgia, venous stasis, leg edema, arthritis, and habitual foot tapping). The symptoms of RLS cause concern, distress, sleep disturbance, or impairment in mental, physical, social, occupational, educational, behavioral, or other important areas of functioning.

The RLS diagnosis is clinically based on the presence of the five international diagnostic criteria (61, 62). The Hening Telephone Diagnostic Interview (HTDI), the Cambridge–Hopkins diagnostic questionnaire for RLS (CH-RLSq), and the RLS diagnostic index (RLS-DI) are useful diagnostic instruments, although not specific for PD patients (61).

If RLS is mild, it can be managed by only lifestyle changes. Therefore, before initiating any pharmacological treatment, it is necessary to evaluate the frequency and duration of symptoms and their impact on the patient’s quality of life. Chronic renal failure, iron, vitamin B12 and folic acid deficiency, serum glucose, and HbA1C need to be investigated in order to exclude secondary forms. The serum ferritin level should be measured, and, if the concentration is <50–75 μg/mL, or if transferrin saturation is less than 20%, supplementation with oral iron is recommended. If oral iron is poorly tolerated or contraindicated, the intravenous administration can also be considered (63). The withdrawal of drugs that potentially exacerbate RLS such as antidopaminergic drugs, antihistamines, and antidepressants (except for bupropion) is also recommended (63).

Treatment of RLS in PD patients has not been evaluated in controlled studies. DAs have proven effective for RLS. The lowest possible cumulative daily dose is recommended to prevent augmentation, which is a side effect characterized by an overall increase in RLS symptoms severity during therapy (63, 64). To prevent such augmentation, long-acting DAs should be preferred to short acting ones. Alternatively, α2δ ligands (pregabalin 150–450 mg/day; gabapentin 900–2.400 mg/day; enacarbil 600–1.800 mg/day) are useful. Dizziness, somnolence, and fatigue are common α2δ ligands side effects. In resistant cases, low doses of opioids such as long-acting oxycodone or methadone should be considered, except for patients with high risk of addiction or with preexisting severe constipation, sleep apnea syndrome, or prolonged QTc (63). Finally, patients may obtain temporally relief by rubbing or massaging the affected limbs, bathing in hot or cold water, physical activity, or distracting themselves with mental exercises (for example, reading an interesting book at the onset of the symptoms) (65).

#### Periodic Limb Movements (PLMs)

Periodic limb movements are stereotyped and repetitive movements affecting the limbs, in particular, the legs, which can cause non-restorative sleep (11). More than 15 movements per hour of sleep are considered pathological, although the prevalence of PLMs increases with age also in the normal population. In elderly, the prevalence of PLMs is estimated between 25 and 58% (11, 66).

Subjects with PLMs may or may not be aware of the movements that are instead reported by the bed partner. Some authors reported a possible link between the presence of PLMs and frequent awakenings, daytime sleepiness, fatigue or, more rarely, EDS (67–69). PLMs are often associated with RLS, narcolepsy, or sleep apnea (70). Eighty percent or more of all RLS patients have PLMs (71–73).

It is not clear, if prevalence of PLMs in PD is higher than in the general population (74–77). Age, secondary causes such as hyposideremia and the loss of dopaminergic cells may explain the link existing between PLMs and PD (78, 79). Antidepressants, neuroleptics, and lithium could be other potential inducing factors (11).

Before starting a specific therapy for PLMs, it is important to evaluate their clinical impact because PLMs could be only an incidental videopolysomnography (VPSG) finding that does not disturb sleep. There are few studies directly assessing the therapy for PLMs in PD and the principal information regarding PLMs treatment comes from RLS patients (63) In RLS patients, randomized placebo-controlled studies have established the efficacy of DAs for both improvement of RLS symptoms and reduction in PLMs. In a cross-sectional study on 19 untreated PD patients, the introduction of DAs therapy almost totally suppressed PLMs (80).

### Parasomnias

#### NREM Parasomnias

NREM parasomnias are undesired events characterized by an incomplete arousal from NREM sleep and include confusional arousals, sleepwalking, and sleep terrors (Table 3) (11). The prevalence of NREM parasomnias in PD is not clear. A questionnaire study on 661 PD patients showed that sleepwalking had a prevalence of 1.8% while night terrors 3.9% (81). In PD patients with RBD, the prevalence of NREM parasomnias (parasomnia overlap disorder) is higher, reaching respectively, 4.3 and 8.7% (81). Somnambulism in PD is associated with higher incidence of depression and advanced PD stage (82). Probably, the neurodegenerative changes of advanced PD influence not only the control of muscle tone but also the mechanisms of state transition (83).

**Table 3:** **Diagnostic criteria for NREM parasomnias (International Classification of Sleep Disorders)**.

Criteria A–E must be met: Recurrent episodes of incomplete awakening from sleep. Inappropriate or absent responsiveness to efforts of others to intervene or redirect the person during the episode. Limited (e.g., a single visual scene) or no associated cognition or dream imagery. Partial or complete amnesia for the episode. The disturbance is not better explained by another sleep disorder, mental disorder, medical condition, medication, or substance use.

The first approach to a patient with NREM parasomnias, independently of its causes, is to secure the bedroom in which the patient sleeps, closing and locking doors or windows, blocking stairways, and removing all potentially dangerous objects. It is also important to reduce all the possible precipitating and predisposing factors such as alcohol consumption, stress, fever, sleep deprivation, sleeping in unfamiliar or noise-exposed bedrooms. If present, it is necessary to treat OSA and reduce or withdraw psychotropic drugs such as phenothiazines, anticholinergic agents, and sedative/hypnotic agents (11). Clonazepam (CNZ) (0.25–2 mg at bedtime) is the first line pharmacologic treatment for NREM parasomnias in adults, but its efficacy has not been proved in randomized controlled trials (84–87). CNZ was effective in 90% of a series of 20 patients with parasomnia overlap disorder (88), but there are no studies in PD patients. Only four sleepwalkers were identified in a set of 165 PD patients. After a 2-year follow-up, two patients have had a spontaneous remission of sleepwalking, one patient responded to topiramate treatment (100 mg/day) and one patient showed a resolution of the episodes after clozapine treatment (25/mg die) (89).

#### REM Parasomnias

##### Nightmares

Nightmares are vivid and unpleasant dreams recurring in REM sleep and causing awakening (Table 4) (11). Their prevalence in PD patients seems to be 17.2%, but these data are only based on questionnaires (81). Dream contents in PD patients seem to be different from controls in particular regarding violence, misfortune, and presence of animals. Some authors explain these contents in relation to the cognitive and frontal impairment of PD patients instead of therapy, mood disorders, hallucinations, and the presence of RBD (90). Traumatic events, stress, use of antidepressants, alpha-agonists, beta-blockers, and cholinergic antagonists are all predisposing and precipitating factors for nightmares (91).

**Table 4:** **Diagnostic criteria for nightmares (International Classification of Sleep Disorders)**.

Criteria A–C must be met: Repeated occurrences of extended, extremely dysphoric, and well-remembered dreams that usually involve threats to survival, security, or physical integrity. On awakening from the dysphoric dreams, the person rapidly becomes oriented and alert. The dream experience, or the sleep disturbance produced by awakening from it, causes clinically significant distress or impairment in social, occupational, or other important areas of functioning as indicated by the report of at least one of the following: Mood disturbance (e.g., persistence of nightmare affect, anxiety, and dysphoria). Sleep resistance (e.g., bedtime anxiety, fear of sleep/subsequent nightmares). Cognitive impairments (e.g., intrusive nightmare imagery, impaired concentration, or memory). Negative impact on caregiver or family functioning (e.g., nighttime disruption). Behavioral problems (e.g., bedtime avoidance, fear of the dark). Daytime sleepiness. Fatigue or low energy. Impaired occupational or educational function. Impaired interpersonal/social function.

The first-line treatment for nightmares is cognitive behavioral therapy, in particular, the imagery rehearsal therapy (IRT), but these techniques have not been systematically tested in PD patients and need to be confirmed. A placebo-controlled study showed that prazosin (9.5 mg/at bedtime) reduced nightmares in 10 Vietnam veterans. Other studies proved prazosin efficacy, but its real effectiveness needs to be tested in larger placebo-controlled trials (92–95). Side effects such as orthostatic hypotension could discourage its use in PD patients.

##### REM Sleep Behavior Disorder

**Figure 4:**
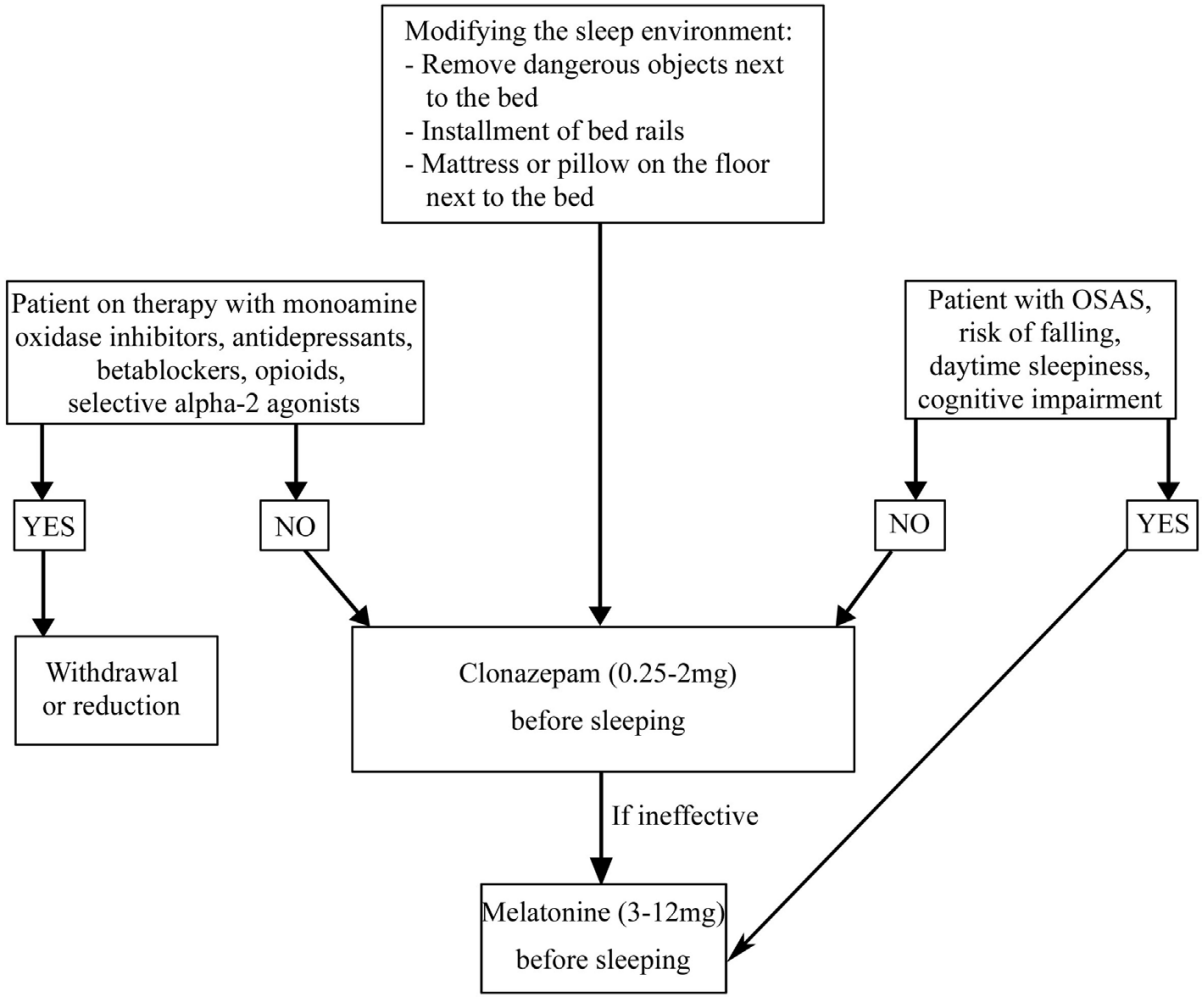
**Treatment algorithm for REM sleep behavior disorder**.

REM sleep behavior disorder is a REM parasomnia characterized by complex, sometimes violent, and dangerous motor behaviors during which the patient acts out the content of his/her dream (Table 5) (11). RBD is present in 30% of patients with PD and often precedes the onset of motor symptoms (1). The association between PD and RBD may be explained considering the brainstem abnormalities in regions that control REM sleep such as the peduncle pontine nucleus and the laterodorsal tegmental nuclei, which are affected during Braak’s stages 1 and 2 (96). Independent of pharmacological therapy, in order to guarantee the patient and bed partner’s safety, securing the bedroom is necessary (Figure 4) (97). It is also necessary to withdraw or reduce drugs potentially causing RBD, such as monoamine oxidase inhibitors, antidepressants, beta blockers (bisoprolol), opioids (tramadol), and centrally acting alpha-agonist hypotensive agents (clonidine) (98–108). If RBD causes sleep disruption or if it influences the patient and bed partner’s safety, pharmacological treatment is indicated (97). CNZ, 0.25–2 mg 30 min prior to bedtime has been a first-line therapy for RBD (97). Several large case series proved the efficacy of CNZ in 87–90% RBD patients with or without PD, also if no randomized placebo-controlled studies have been performed (85, 109–111). Sedation and increased risk of fallings are CNZ possible side effects. CNZ is contraindicated in moderate and severe OSAS (85, 110, 112). The mechanism of action of CNZ is unknown. Considering that it does not suppress REM sleep and it does not influence REM muscle tone, probably it could modify dream contents or inhibit brainstem locomotor pattern generators (113, 114). Recent studies proved that CNZ moderately increases total sleep time, sleep efficiency, NREM sleep stages (except stage 1), and decreases wake after sleep onset (WASO) (115). In PD patients in which CNZ is contraindicated (for example, patient with OSAS, cognitive impairment, and a high baseline risk of falling), melatonin (3–12 mg, before sleeping) could be the treatment of choice. CNZ and melatonin appear comparably effective for RBD symptoms and injury prevention. Melatonin’s favorable safety and tolerability profile is very useful for patients receiving polytherapy and for neurologically impaired RBD patients who are more sensitive to adverse drugs effects (116, 117). A recent meta-analysis of randomized clinical trials suggested that exogenous melatonin, improving sleep quality in patients with neurodegenerative disorders can be considered as a possible mono or add-on therapy in patients with RBD (118).

**Table 5:** **Diagnostic criteria for REM sleep behavior disorder (International Classification of Sleep Disorders)**.

Criteria A–D must be met: Repeated episodes of sleep-related vocalization and/or complex motor behaviors. These behaviors are documented by polysomnography to occur during REM sleep or, based on clinical history of dream enactment, are presumed to occur during REM sleep. Polysomnographic recording demonstrates REM sleep without atonia. The disturbance is not better explained by another sleep disorder, mental disorder, medication, or substance use.

Melatonin mechanisms of action are still not completely known, but its use seems to reduce RBD and decrease muscle tone during REM sleep. Side effects (morning headache, morning sleepiness, and delusions/hallucinations) are usually related to high doses (117, 119, 120). Pramipexole (0.2–1 mg/night), paroxetine (10–40 mg), donepezil (10–15 mg), and rivastigmine (4.5–6 mg) may be effective in some refractory cases, but the evidence of their efficacy is inconclusive due to the lack of randomized controlled trials (97, 121–123). Two studies have been carried out on rivastigmine. Rivastigmine compared to placebo induced a reduction in the frequency of RBD episodes in a pilot placebo-controlled crossover trial on 25 patients with mild cognitive impairment (but without PD). Similar results have been found in a double-blind, crossover trial in 12 patients with PD and RBD resistant to CNZ and melatonin (124, 125). Very limited evidence has been reported for zopiclone, benzodiazepines other than CNZ, yi-gan san, desipramine, clozapine, carbamazepine, and sodium oxybate (97). Ramelteon was effective in RBD in PD patients in two recent open trials, conducted, respectively, in 24 and 12 PD patients, but these promising results could be confirmed in larger population and for longer follow-up. Daytime sleepiness, nausea, delirium, giddiness, and worsening of constipation are possible ramelteon side effects (40, 126).

### Sleep-Related Breathing Disorders

#### Obstructive Sleep Apnea

Obstructive sleep apnea is characterized by snoring and repetitive episodes of complete (apnea) or partial (hypopnea) upper airway obstruction occurring during sleep. The patient typically complains of lack of breath, gasping or choking, insomnia, non-restorative sleep, and EDS (11). The prevalence of OSA in PD is highly controversial, widely ranging from 20 to 60% (5, 7, 127–129). Although middle-elderly age, fluctuating respiratory muscle coordination, autonomic dysfunction, and reduced respiratory drive may justify a higher prevalence of OSA in PD patients, recent studies demonstrated that OSA’s prevalence in PD is the same of the general population. At the same time, sleep fragmentation and intermittent hypoxemia due to OSA may contribute to and worsen PD (4, 130–136).

The gold standard therapy for OSA is the continuous positive airway pressure (CPAP), which normalizes nocturnal respiration improving nighttime oxygenation, sleep architecture (particularly deepening sleep), and daytime sleepiness (136). The control of body weight and sleeping on one’s side during the night could be useful. In this case, to relieve the difficulties of turning around, adapted physiotherapy and increased dopaminergic treatment at night by extra nocturnal doses or sustained release drugs may improve OSA (137). Recently, mandibular advancement devices have been shown to be similarly beneficial to CPAP in improving OSA (138). There are contradictory reports on the modulation of respiratory function by antiparkinsonian treatment and the real effect of DAs remains unknown (139, 140). DAs seem to enhance the risk of central SDs of breathing (141). On the contrary, bedtime long-acting LD seems to improve OSA in PD patients as shown in a polygraphic study in 57 patients, even if the real mechanism of action of LD has not been proven (142).

## Diurnal SDs

### Excessive Daytime Sleepiness

Excessive daytime sleepiness, first described as “sleep attack” characterized by sudden and irresistible overwhelming sleepiness without awareness of falling asleep (143), is more often reported as sleepiness with prodromes such as tearing or yawning (144, 145) (Table 6). EDS is present in 21% of patients with PD and increases with disease progression (1). Age, sleep–wake cycle alteration related to PD, PLM, daytime immobility, and dopaminergic medications may contribute to EDS (4, 8, 146–148). Moreover, a study on 10,053 drug-naive patients with early PD proved that patients who developed EDS after 5 years of therapy were those with higher sleepiness at the baseline (149). The Multiple Sleep Latency Test (MSLT), the Maintenance of Wakefulness Test (MWT), and the Epworth Sleeping Scale (ESS) may be inadequate to detect sleepiness in PD patients (4, 5, 8, 146, 147, 150). The Inappropriate Sleep Composite Score (ISCS) instead has a higher specificity of predicting PD patients with increased risk of accidents with motor vehicle due to fall asleep while driving (151).

**Table 6:** **Diagnostic criteria for excessive daytime sleepiness (International Classification of Sleep Disorders)**.

Criteria A–D must be met: The patient has daily periods of irrepressible need to sleep or daytime lapses into sleep occurring for at least 3 months. The daytime sleepiness occurs as a consequence of a significant underlying medical or neurological condition. If an MSLT is performed, the mean sleep latency is ≤8 min and fewer than two sleep onset REM periods are observed. The symptoms are not better explained by another untreated sleep disorder, a mental disorder, or the effects of medications or drugs.

**Figure 5:**
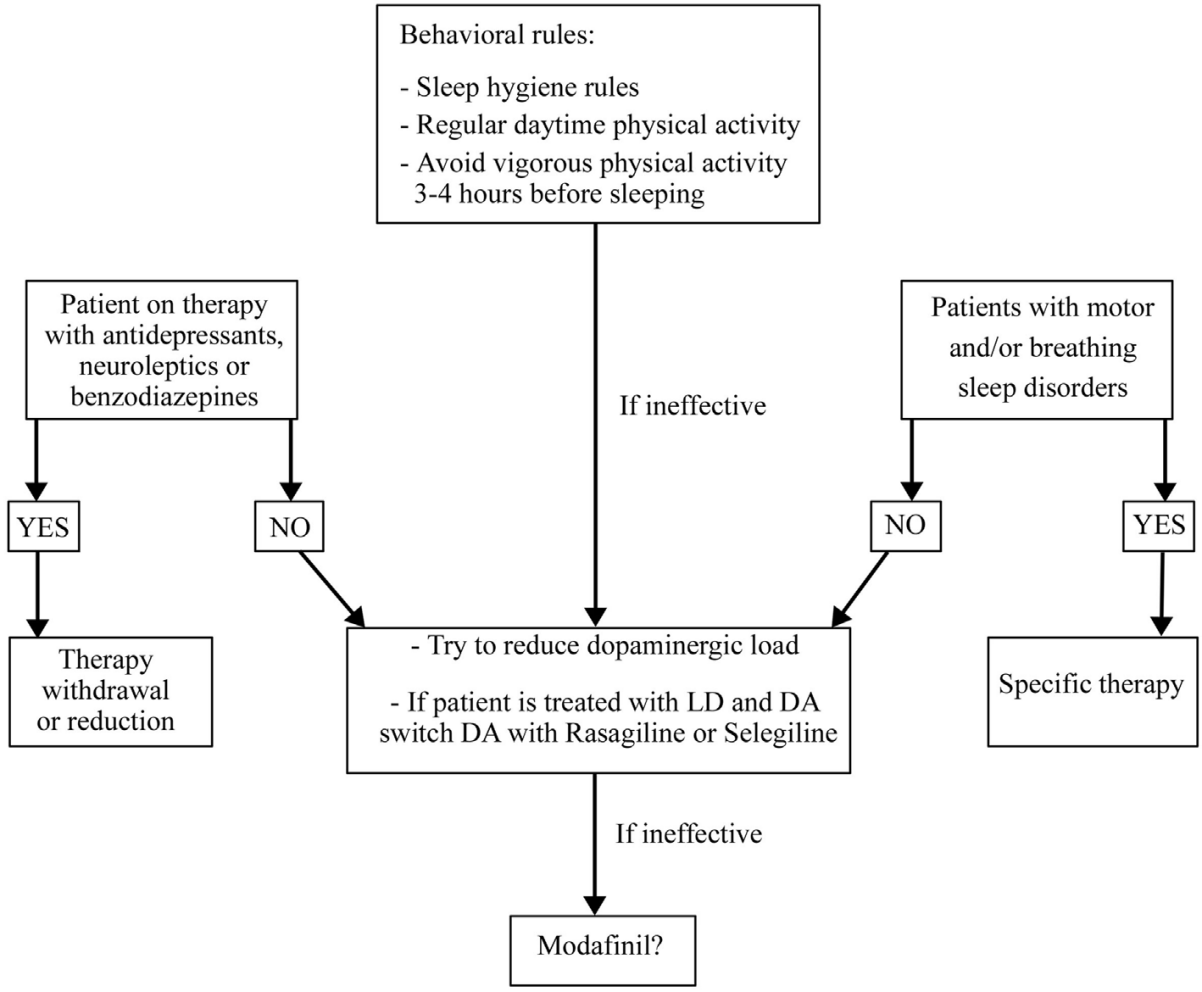
**Treatment algorithm for excessive daytime sleepiness**.

Treatment of EDS in PD is a challenge. First of all, it is necessary to identify and treat any possible SDs that could disrupt nocturnal sleep and to withdraw or reduce any possible drugs causing hypersomnia such as antidepressants, antipsychotics, or sedatives. At the same time, it is important to educate patients to apply the sleep hygiene rules (Figure 5). All DAs cause more EDS than LD without differences between drugs but with a direct relationship with drug dose (152, 153). Combination therapy with LD and DAs shows the highest risk of EDS (154). Instead, selegeline, amantadine, and entacapone had no influence on EDS (155, 156). The association between orally dispersible selegiline and DAs may reduce or resolve EDS (150, 153, 154, 156). If the above strategies do not improve EDS, the use of stimulating drugs such as modafinil (100–400 mg/day) should be a solution. It seems to improve patients’ perception of wakefulness without any objective confirmation (41). Two randomized, double-blind, crossover, placebo-controlled trials on small groups of PD patients with EDS showed that modafinil 100–200 mg/day after 2 weeks of treatment, improved the ESS scores but not sleep latency on the MWT (157). On the other hand, a double-blind, placebo-controlled parallel design trial on 40 PD patients treated with modafinil 200–400 mg/day for 4 weeks showed no improvement of ESS scores (158). A recent review and meta-analysis of pharmacological interventions for EDS in Parkinson’s disease concluded that modafinil improves daytime sleepiness of PD patients also if the significance of this improvement was not robust to all sensitivity analyses (42). Possible adverse events related to modafinil therapy are headache, dry mouth, dizziness, nausea, nervousness, insomnia, and generalized itching (42, 159, 160). These side effects seem to be mild and decrease with dose reduction (161). Nocturnally administered sodium oxybate 3–9 g/night in two split doses (at bedtime and 4 h later) may improve EDS and fatigue in PD, but further and larger studies are needed to prove its efficacy and safety considering also its depressant function on the respiratory centers and its abuse potential. Only one multicenter, open-label, polysomnographic study tested the efficacy of sodium oxybate after 12 weeks of therapy on 30 PD patients demonstrating an improvement of ESS, Pittsburgh Sleep Quality Inventory score, Fatigue Severity Scale score, and an increase in slow-wave sleep (162). Methylphenidate (1 mg/kg three times daily, for 3 months) was effective after a night of LD withdrawal and after an acute administration of LD, in reducing ESS in 17 patients with advanced PD (163). Unlike modafinil and sodium oxybate, which did not influence motor PD symptoms, methylphenidate improved them and, in particular, gait (163). Finally, caffeine (200 mg/bid) seems to improve motor symptoms after 3 weeks of treatment, in the absence of any real influence on EDS in a randomized, double-blind, crossover, placebo-controlled, multicenter trial on 61 patients (164).

## Deep Brain Stimulation (DBS)

Deep brain stimulation is an established therapy for PD. The effects of DBS on sleep have been proved in many studies, but it is not clear if DBS modifies sleep directly or indirectly improving PD symptoms. Subthalamic nucleus (STN)-DBS improves PSQI and PDSS scores with contrasting data on objective sleep parameters evaluated by polysomnography in small cohorts of patients (165–174). Fifteen PD patients who underwent STN-DBS showed an improvement in total sleep time and sleep efficiency with a decrease in WASO and arousal index assessed by VPSG. This study found no link between sleep modifications and motor improvement (170). Two other studies, respectively, in 10 and 11 patients, did not find any significant changes in sleep efficiency after STN-DBS, although an increase in uninterrupted sleep and REM sleep and a reduction in arousal index has been found (168, 171). Another study conducted in 10 patients showed also an increase in slow-wave sleep after STN-DBS, without any changes in sleep latency and number of awakenings (175, 176). DBS seems effective in reducing sleep onset insomnia as documented in two studies that evaluated initial insomnia with PDSS in 5 and 10 PD patients (167, 173). One of these studies, in particular, using stimulation of pedunculopontine nucleus demonstrated also an improvement in maintenance insomnia and daytime sleepiness (173). Another study showed an improvement in RLS symptoms in 16 patients but also an improvement in maintenance insomnia and EDS, after STN-DBS (166). On the contrary, other studies did not show any improvement in EDS at the ESS (177–179).

The effects of STN-DBS on RBD have been evaluated in a study on 90 PD patients among which 47 were affected by RBD ascertained by clinical history assessment. After STN-DBS, RBD developed *de novo* in 16 patients within 1 year and persisted in other patients (180). Other studies did not report any increase nor improvement in RBD symptoms after DBS, but the cohorts examined in these studies were smaller (168, 175, 176). A study on 195 patients who underwent STN-DBS demonstrated the new onset of RLS in 11 patients (181). The same results have been found in 6 out of 31 PD patients after STN-DBS (182). Other studies have instead reported a reduction in RLS symptoms after surgery (166, 183).

Subthalamic nucleus-DBS seems not to influence PLMs (168, 171) nor the occurrence of apnea–hypopnea (168, 175).

## Conclusion

Sleep disorders in PD are frequent. Understanding sleep problems in PD and their treatment is challenging, but it will help to improve the management of PD, improving the quality of life in these patients. An accurate clinical and diagnostic assessment is mandatory before starting the treatment. The first objective to achieve is to understand if SDs are a primary or secondary disorder and if behavioral rules or a dose modification of the ongoing therapy could improve SDs. Many different treatments options are now available to treat SDs in PD but further and larger randomized controlled trials are needed to confirm their efficacy and to solve these conflicting data. A better understanding of the relationship between sleep–wake regulation and PD could guide future research and facilitate the management of the disease.

## Author Contributions

GL: drafting of manuscript, critical revisions, and final approval. GC-B: critical revisions and final approval. LS: critical revision and final approval. GG: critical revisions and final approval. AC: critical revisions and final approval. PC: critical revisions and final approval. FP: drafting of manuscript, critical revisions, and final approval.

## Conflict of Interest Statement

GL received honoraria for participation in clinical trial as sub-investigator from UCB Pharma; PC received honoraria for speaking engagements or consulting activities from Allergan Italia, Lundbeck Italy, UCB Pharma S.p.A, Chiesi Farmaceutici, AbbVie srl, Eli Lilly and Company, Zambon; FP received honoraria for speaking engagements or consulting activities from Sanofi and Bial. The other authors declare no conflict of interest.
